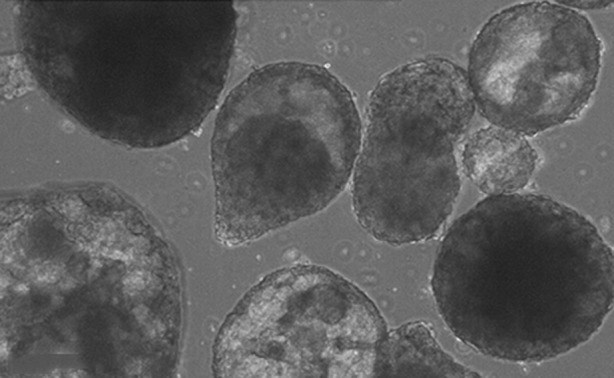# Modelling DMD-associated cardiomyopathy in patient-derived iPSCs

**Published:** 2015-05-01

Duchenne muscular dystrophy (DMD) is a genetic muscular disorder characterised by progressive muscular weakness and wasting, with cardiac complications (such as dilated cardiomyopathy) and respiratory muscle failure arising in the late stage. To investigate mechanisms of dilated cardiomyopathy in DMD, Lei Yang and collaborators derived cardiomyocytes (CMs) from DMD-patient-specific induced pluripotent stem cells (iPSCs). Compared to control cells, DMD iPSC-CMs exhibited elevated cytosolic calcium, mitochondrial damage and increased cell apoptosis. To further dissect the mechanisms underlying these alterations, the authors performed transcriptional and translational analyses and identified a mitochondrially initiated molecular cascade – which involves CASPASE3 (CASP3) activation – as being responsible for the increased apoptosis in DMD iPSC-CMs. Notably, the application of the membrane sealant Poloxamer 188 could prevent calcium overload and CASP3 activation, significantly reducing apoptosis in these cells. Thus, the authors established a useful *in vitro* system to disclose mechanisms of cardiomyopathy in DMD and to identify molecular targets that could be pharmacologically manipulated. **Page 457**

**Figure:**